# Genetic Polymorphisms on *OPRM1, DRD2, DRD4*, and *COMT* in Young Adults: Lack of Association With Alcohol Consumption

**DOI:** 10.3389/fpsyt.2020.549429

**Published:** 2020-12-07

**Authors:** Patrick Chung, Warren B. Logge, Benjamin C. Riordan, Paul S. Haber, Marilyn E. Merriman, Amanda Phipps-Green, Ruth K. Topless, Tony R. Merriman, Tamlin Conner, Kirsten C. Morley

**Affiliations:** ^1^Discipline of Addiction Medicine, Central Clinical School, Faculty of Medicine and Health, University of Sydney, Sydney, NSW, Australia; ^2^Psychological Medicine, Central Clinical School, Faculty of Medicine and Health, University of Sydney, Sydney, NSW, Australia; ^3^Drug Health Services, Royal Prince Alfred Hospital, Camperdown, NSW, Australia; ^4^Department of Biochemistry, University of Otago, Dunedin, New Zealand; ^5^Department of Psychology, University of Otago, Dunedin, New Zealand

**Keywords:** alcohol consumption, genetics, risky (problem) drinking, emerging adult, OPRM1 A118G, DRD2

## Abstract

**Background:** Risk behaviors for young adults such as alcohol use are associated with increased risk of morbidity and mortality. Patterns of risk behavior may be genetically determined and vary between genders. Previous studies in both young adults and heavy drinking adult samples have demonstrated that some genotypes, such as *OPRM1* A118G, *COMT* Val158Met and *DRD2* Taq1A and *DRD4* C52IT, may predict addictive behaviors including alcohol consumption and impulsivity, although results have been mixed.

**Methods:** This study aimed to investigate the predictive relationship of these four single nucleotide polymorphisms (SNPs) prospectively on student patterns of drinking using a micro-longitudinal daily diary design in a sample of 628 young adults ages 18–25 of predominantly of European ethnicity. Linear mixed models were used to examine the effect of SNPs on the number of drinks per drinking session with gender as a moderating variable.

**Results:** There were no main effects for genotype on alcohol consumption, nor for gender × genotype for any of the SNPs. There was a trend for an effect of the *DRD2* Taq1A on the number of drinks per drinking day and for the interaction of gender and *DRD2* Taq1A on the number of drinks per drinking day.

**Conclusion:** These findings suggest that the *DRD2* Taq1A*, OPRM1* A118G, *DRD4* C521T, or *COMT* Val158Met polymorphisms, are not associated with alcohol consumption in young adults, although there may be a relationship between *DRD2* Taq1A and alcohol consumption in young adult males.

## Introduction

Risk behaviors such as regular and heavy alcohol use are often initiated during the teenage years and continue on into early adulthood (1). Regular alcohol use and binge drinking are risk behaviors of particular concern and are associated with increased risk of morbidity and mortality (2). In recent years, certain genes and single nucleotide polymorphisms (SNPs) have become of interest due to their possible roles in the development of addiction and risk behaviors. Some of these genes and their SNPs include *DRD2* Taq1A, *DRD4* C521T, *COMT* Val158Met, and *OPRM1* A118G.

The *OPRM1* gene codes for the μ-opioid receptor and is associated with pain modulation (3). The G allele of the A118G has been widely studied with some studies reporting an association with cravings for alcohol in adult heavy drinkers (4) and alcohol misuse in adolescents (5). One study observed appetitive behaviors for alcohol were associated with the G allele in a sample of heavy drinking young males (6). However, an ethnic-specific meta-analysis by Chen et al. (7) demonstrated that the A118G SNP is associated with alcohol dependence in Asian adults but not among Caucasian adults. Although potentially underpowered, one recent study in a sample of 106 social drinkers with European ancestry demonstrated no association between *OPRM1* rs1799971 genotype and alcohol consumption or sensitivity (8).

DRD2 and DRD4 are the genes that code for the D2 and D4 dopamine receptors, respectively. Two single nucleotide polymorphisms (SNP) associated with dopamine receptors *DRD2* (Taq1A) and *DRD4* (521CT) have been studied in relation to addictive behaviors. The A1 allele of the *DRD2* (Taq1A) polymorphism has been associated with reduced expression of the D2 dopamine receptor (9). Meta-analyses have demonstrated an association between the Taq1A polymorphism and alcohol dependence (10, 11) albeit with a moderate effect size and potential for some publication bias influenced by racial ancestry (11). The *DRD4* C521T SNP has been suggested to be related to novelty-seeking behavior (12–14) although there have been no studies to date examining any relationship with alcohol. Another polymorphism in the variable number of tandem repeat (VNTR) polymorphism of *DRD4* has been observed to moderate the effect of alcohol consumption on social bonding in young adult social drinkers (15). There have been no studies examining the *DRD4* C521T SNP.

The COMT gene codes for the catechol-o-methyltransferase enzyme that degrades neurotransmitters (16). The Met (A) allele of the Val158Met SNP is associated with reduced enzymatic activity of catechol-o-methyltransferase, which has been suggested to have possible implications in neuropsychiatric disorders (17) in addition to both gambling and drinking problems in non-clinical samples (18) and impulsive behavior in young adults (19). Studies examining the association of the Val158Met SNP with alcohol problems in adults have reported conflicting results. For example, one study has associated the COMT Met/Met genotype with greater alcohol use in social drinkers (20) while others have found no association with alcohol dependence (21). Additionally, one study found sex-specific associations for high-activity COMT and alcohol dependence, seen in male alcohol dependent participants only (22). It is possible that the effect of the Met/Met genotype on drinking behavior is more easily observed in social drinkers rather than in association with a clinical diagnosis, and/or is associated with risky decision making.

Taken together, the literature regarding the above candidate genotypes and alcohol consumption is somewhat inconsistent, which may be due to differences across studies including varying ethnicity, clinical heterogeneity, and sample selection such as studies of young social drinkers vs. adult dependent drinkers. This study thus aims to extend this literature by investigating the relationship of the above-mentioned candidate genotypes on prospective alcohol consumption across a 2 week period in a sample of university students.

## Materials and Methods

### Participants and Procedure

Participants were 628 young adults aged between 18 and 25 years of age studying at the University of Otago, New Zealand (see Table 1 for demographics). The data were collected in 2011 and 2012 as part of the Daily Life Study, and carried out in accordance with the recommendations and approval of the University of Otago Human Ethics Committee (10/777). All participants gave written informed consent in accordance with the Declaration of Helsinki by the 18th WMA General Assembly. Students were recruited predominantly from psychology courses and additional students were recruited through student job search, flyers, and social networking sites. Psychology students were reimbursed with class credit and were reimbursed for costs involved. Those recruited elsewhere were given renumeration. All renumeration was scaled based on their participation within the experiment.

**Table 1:** Sample demographics and descriptive statistics.

**Characteristic**	
*N*	628
Male, *N* (%)	237 (37.7%)
Female, *N* (%)	391 (62.3%)
Age (years)	19.81 (3.159)
Year at university	2.01 (0.940)
Drinks per drinking day	7.5 (4.69)
Number of sessions	3 (range 2–8)
European/Caucasian	496 (79%)

*Data represent mean (standard deviation) unless otherwise stated. Drinks per drinking day and number of sessions were for the entire sample across the 13 day testing period*.

Participants completed a baseline survey with demographic questions and trait measures in private cubicles, completed an Internet daily diary survey each day between 3 and 8 pm for 13 consecutive days, gave a non-fasting 22 ml venous blood sample at an on-campus clinic (4 people gave a saliva sample), and completed a final survey. Participants were asked in the daily diary how many standard drinks they had consumed the night before and were given a guide to estimate it.

### Genotyping

Genotypes for the *OPRM1* (A118G), *DRD2*_taq1A (ANKK1-Taq1a variant), *DRD4*_521(C521T), *COMT* (Val158Met) were obtained from blood (99%) or saliva samples. DNA was extracted from peripheral white blood cells using a guanidine-HCl-based procedure with chloroform extraction (23) and genotyped for the rs1799971, rs1800497, rs1800955, rs4680 genotypes using a Life Technologies TaqMan 5 nuclease assay (probe id C_3290335_10) and performed according to manufacturer's instructions. Fourteen percent of samples were repeated with 100% concordance in genotype. Hardy Weinberg Equilibriums (HWEs) were calculated using the Court lab–HW calculator.

### Data Analysis

We aimed to determine the association between each genotype and the number of drinks per drinking day over the 13 days when participants were surveyed. A drinking day or session was counted as >0 standard drinks in the preceding day. We included data only for the participants that reported at least one drinking session and those that had completed at least 50% of the surveys.

Multilevel modeling was conducted in SPSS for Windows, version 25, employing linear mixed effects modeling. A random intercept-only model was fitted with participant as the random factor and dependent variable of number of drinks per drinking day. Separate models were conducted per genotype, which were added as fixed effects, whereby the major-allele homozygote was the reference genotype. As the sample was predominantly female (62.3%), gender was included as a control fixed factor main effect and a moderator with genotype. Ethnicity in this sample was also heavily skewed toward European Caucasian (79%) and was likewise added a control fixed factor (European Caucasian or Non-European Caucasian). The *p*-value was set to <0.0125 using a Bonferroni correction (0.05 divided by 4 genotypes) to adjust for multiple hypothesis testing. Based on an estimated sample prevalence of G- for OPRM1 for AUD vs. non AUD in young social drinkers (51.9 and 16.3%) with a medium magnitude effect size, estimated from (5), with a final sample of 628, we had more than sufficient power to detect a medium size difference between the homozygotes at α = 0. 0125.

## Results

### Sample Demographics and Drinking Measures

Table 1 summarizes the demographic and clinical characteristics of the sample. We sampled *N* = 628 university students who were predominantly female (62.3%, *N* = 391) and predominantly of European ethnicity (79%, *N* = 496; Asian = 9.9%, *N* = 62; Pacific Islander = 3.3%, *N* = 21; Indian = 3%, *N* = 19; Middle Eastern = 1.1%, *N* = 7, Mixed = 1.6%, *N* = 10, Other = 2.1%, *N* = 13). The average number of surveys completed was 11.41 (SD = 1.945) out of 13, which was an 88% response rate. Participants consumed an average 7.5 standard drinks (SD = 4.69) per drinking day, with a overall median of 3 drinking sessions (range: 2–8).

### Effects of Polymorphism Variants on Drinking

Genotype distributions per polymorphism are depicted in Table 2 and mixed model fixed effects tests are displayed in Table 3 and full models are displayed in the Supplementary Tables 1–4.

**Table 2:** Genotype distributions of each polymorphism per variant.

**Genotype**	**Variant**		
*OPRM1 A118G*	GG = 12	GA = 97	AA = 358
*DRD2 Taq1A*	A2/A2 = 36	A1/A2 = 208	A1/A1 = 381
*DRD4 C521T*	CC = 123	CT = 275	TT = 224
*COMT Val158Met*	GG = 168	GA = 296	AA = 163

*Genetic polymorphisms and alcohol use*.

**Table 3:** Tests of fixed effects for overall models of each polymorphism.

	***OPRM1 A118G***			***DRD2 Taq1A***			***DRD4 C521***			***COMT Val158***		
**Fixed effects**	***df***	***F***	***p***	***df***	***F***	***p***	***df***	***F***	***p***	***df***	***F***	***p***
Variant	1,275.72	0.76	0.383	2,279.46	2.44	0.089	2,276.21	0.35	0.705	2,280.08	0.09	0.910
Ethnicity	1,283.24	1.53	0.218	1,270.46	1.54	0.216	1,277.53	0.82	0.365	1,278.94	0.88	0.348
Gender	1,274.02	20.8	<0.001	1,286.78	24.35	<0.001	1,274.91	27.73	<0.001	1,280.67	24.21	<0.001
Variant *Gender	1,273.46	0.04	0.851	2,280.51	2.86	0.059	2,275.25	0.58	0.559	2,279.56	0.68	0.506

*All variant variables were dummy coded into three levels for the allele variants except for OPRM1 A118G which pooled GG and G/A*.

*Models for each polymorphism including the specific variant tests can be observed in the Supplementary Tables 1–4*.

#### OPRM1 A118G rs1799971

Genotype distributions of the *OPRM1* A118G polymorphism were GG (*N* = 12), GA (*N* = 97), AA (*N* = 358), which were in HWE (χ ^2^= 2.92, *p* = 0.09). Fixed effects tests revealed males consumed significantly more drinks per day than females (*p* <0.001), but there were no significant effects of gene variants, ethnicity, or genotype and gender interaction (p's > 0.218).

#### DRD2 Taq1A rs1800497

*DRD2* Taq1A polymorphism distributions were A1/A1 (*N* = 381), A1/A2 (*N* =208), A2/A2 (*N* =36), which were in HWE (χ^2^ = 1.140, *p* = 0.286). Fixed effects tests demonstrated males consumed significantly more drinks per drinking day than females (*p* <0.001). There were trends for *DRD2* Taq1A variant differences (*p* = 0.089), and stronger trend toward an interaction of polymorphism variant and gender interaction (*p* = 0.059), with fixed effects estimates showing a significant interaction with gender and comparison between homozygous variants A1/A1 vs. A2/A2 (*p* = 0.048). Sidak-adjusted pairwise comparisons of these variants stratified by gender revealed males with the A2/A2 variant (estimated mean = 13.7) drank significantly more per day than males with A1/A1 variant (estimated mean = 8.58) (*p* = 0.021), with no effect seen for females or other comparisons (*p*'s > 0.113). No effect for ethnicity was seen (*p* = 0.216).

#### DRD4 C521T rs1800955

*DRD4* C521T polymorphism distributions were CC (*N* = 123), CT (*N* = 275), TT (*N* = 224), which were did not deviate from HWE (χ^2^ = 5.243, *p* = 0.022, *p* = 0.09 corrected). Fixed effects tests again demonstrated males consumed significantly more drinks per drinking day (*p* <0.001). However, no main effects of ethnicity, gene variant, or variant by interaction effects were observed related to number of drinks per drinking day (*p*'s > 0.365).

#### COMT Val158Met rs4680

Genotype distributions of the *COMT* Val158Met polymorphism were GG (*N* = 168), GA (*N* = 296), AA (*N* = 163), which were in HWE (χ^2^ = 1.9495, *p* = 0.163). A significant gender effect was seen with males consuming more drinks per drinking day (*p* <0.001), but no significant main effects for ethnicity, variant, or variant and gender interaction upon number of drinks per drinking day were seen (*p*'s > 0.348).

## Discussion

No evidence of main effects of gene variants upon consumption per drinking day were seen in this sample of young adult social drinkers. There were also no significant gene x gender interactions found for *OPRM1* A118G, *COMT* Val158Met and *DRD4* C521T SNPs. There was a trend for an effect of the *DRD2* Taq1A on the number of drinks per drinking day and for the interaction of gender and *DRD2* Taq1A on the number of drinks per drinking day.

An association of *DRD2* Taq1A on alcohol consumption and presence of alcohol dependence has been previously reported in the literature. One meta-analysis demonstrated that positivity of the A1 allele was associated with a greater odds of alcohol dependence (10). In the current study we observed two trends regarding *DRD2* Taq1A rs1800497 on alcohol consumption worth noting including a main effect for *DRD2* Taq1A and also for an interaction between gender and the *DRD2* Taq1A genotype. Our results are somewhat inconsistent with previous literature given that we demonstrated that a trend for an association between A1/A1 and lower levels of alcohol consumption in males relative to those with A2/A2, whereas there were no differences observed among females. Gender differences have also previously been reported in relation to the *DRD2* Taq1A rs1800497 SNP, however in these studies, alcohol dependent males demonstrated a higher proportion of A1 allele than females, and healthy controls (24, 25). It is possible that the predictive role of *DRD2* Taq1A on drinking over time in a sample of young adult social drinkers may differ to an association study examining presence vs. absence of alcohol dependence whereby heavy drinking patterns have already been established. This may be due to different proximal factors for drinking behavior such as varying drinking motives that influence the gene and environment relationship. For example, important drinking motives for college students include social (drinking to improve parties or gatherings), and conformity (drinking due to social pressure or a need to fit in) that may not be present for older adults (26).

The null findings for the *OPRM1* A118G genotype on alcohol consumption are worth noting. Although the *OPRM1* A118G genotype has been widely studied with many studies reporting an association with alcohol dependence, meta-analyses concluded no association of the genotype with a risk of alcohol dependence in Caucasian samples (7, 27). A more recent study reported no association of the *OPRM1* A118G genotype on alcohol consumption or sensitivity in social drinkers of European ancestry in a human laboratory testing paradigm (8). In the current study, we employed multi-level linear mixed modeling to examine the relationship between *OPRM1* A118G genotype and alcohol consumption, our results are consistent with emerging literature suggesting the association of the *OPRM1* A118G genotype with heavy drinking may not be strong in Caucasian samples. Moreover, similar to the above, our sample consists of young adult social drinkers such that the results, while consistent with the latter described human laboratory study (8), may not extend to studies examining association with older adults with alcohol dependence.

## Limitations

While the goal of the current study was to investigate the relationship of the above-mentioned candidate genotypes on prospective alcohol consumption in young adults, there was no assessment and identification of alcohol use disorder in this population. This limited our capacity to determine the specific role of these genotypes on drinking behavior associated with moderate to severe alcohol use disorder. Furthermore, it is possible that we did not have sufficient power to detect small effect sizes regarding associations with *DRD4* C521T, *DRD2* Taq1A, *COMT* Val158Met and *OPRM1* A118G and thus we encourage replication in larger samples.

## Conclusion

Our findings suggest that the *DRD2* Taq1A*, OPRM1* A118G, *DRD4* C521T, or *COMT* Val158Met polymorphisms, are not associated with alcohol consumption in young adults, although there may be a relationship between *DRD2* Taq1A and alcohol consumption in young adult males. Further longitudinal research is required to understand the possible interactions between gender and *DRD2* Taq1A on alcohol consumption in young adults.

## Data Availability Statement

The dataset presented in this article is not readily available because participants in the Daily Life Study (run between 2011 and 2014) did not consent for their data to be publically available. Requests to access the dataset should be directed to TC at tamlin.conner@otago.ac.nz.

## Ethics Statement

The studies involving human participants were reviewed and approved by University of Otago Human Ethics Committee (10/777). The patients/participants provided their written informed consent to participate in this study.

## Author Contributions

TC conceived the Daily Life Study and led collection of the behavioral data. TM led the genetics analysis. KM, WL, PC, and BR conceived the paper idea. WL performed the statistical analysis with assistance from PC and BR. PC and KM wrote the article with assistance from WL. All authors read and reviewed the final manuscript.

## Conflict of Interest

The authors declare that the research was conducted in the absence of any commercial or financial relationships that could be construed as a potential conflict of interest.
