# Supplementary material for: Genetic Polymorphisms on OPRM1, DRD2, DRD4, and COMT in Young Adults: Lack of Association With Alcohol Consumption
Source: Front Psychiatry. 2020 Dec 7;11:549429. doi: 10.3389/fpsyt.2020.549429 (PMC7750453; doi:10.3389/fpsyt.2020.549429)
Supplement: Supplementary file 1 [file Table_1.docx]

**Genetic polymorphisms on *OPRM1, DRD2, DRD4* and *COMT* in young adults: lack of association with alcohol consumption**

**^1^Patrick Chung+, MD, Warren B Logge+, PhD, Benjamin C. Riordan PhD ^2^, Paul S Haber PhD ^1,3^, Marilyn E Merriman^4^, Amanda Phipps-Green^4^, Ruth K Topless^4^, Tony R. Merriman^4^, Tamlin Conner^5^, Kirsten C Morley PhD+^1*^.**

1. Discipline of Addiction Medicine, Central Clinical School, Faculty of Medicine and Health,

University of Sydney, NSW, Australia.

1. Psychological Medicine, Central Clinical School, Faculty of Medicine and Health, University of

Sydney, NSW, Australia.

1. Drug Health Services, Royal Prince Alfred Hospital, NSW, Australia.
2. Department of Biochemistry, University of Otago, Dunedin, New Zealand.
3. Department of Psychology, University of Otago, Dunedin, New Zealand.

+ equal contribution

*** Correspondence:**

**Kirsten Morley:** kirsten.morley@sydney.edu.au

Discipline of Addiction Medicine, Faculty of Medicine and Health, University of Sydney, NSW, Australia.

**Supplementary Material**

*Supplementary Table 1. Overall model for* OPRM1 A118G

| Fixed Effects | Estimate | SE | 95% CI | t | p |
| --- | --- | --- | --- | --- | --- |
| Intercept | 5.81 | 0.86 | 4.11 - 7.51 | 6.72 | <.001 |
| *OPRM1 A118G GG,G/A* | 0.48 | 0.95 | -1.4 - 2.35 | 0.5 | .618 |
| *OPRM1 A11 AA* ^a^ | - | - | - | - | - |
| European/Caucasian | 1.02 | 0.83 | -0.61 - 2.64 | 1.23 | .218 |
| Non-European/Caucasian ^a^ | - | - | - | - | - |
| Gender: Male | 2.91 | 0.6 | 1.73 - 4.1 | 4.84 | <.001 |
| Gender: Female ^a^ | - | - | - | - | - |
| *OPRM1 A118G GG,G/A* * Gender  b | 0.25 | 1.33 | -2.37 - 2.87 | 0.19 | .851 |

| Random Effects | Variance | SE |
| --- | --- | --- |
| Participant (Intercept) | 12.45 | 1.74 |
| Residual | 21.36 | 1.28 |

^a^ Reference category for main effect level comparisons.

^b^ Reference categories for interactions are as indicated for main effects.

*Note*. *OPRM1 A118G GG & G/A* are pooled.

*Supplementary Table 2. Overall model for* DRD2 Taq1A

| Fixed Effects | Estimate | SE | 95% CI | t | p |
| --- | --- | --- | --- | --- | --- |
| Intercept | 5.9 | 1.7 | 2.55 - 9.25 | 3.46 | .001 |
| *DRD2 Taq1A A1/A2* | -0.06 | 1.83 | -3.66 - 3.54 | -0.03 | .973 |
| *DRD2 Taq1A A1/A1* | 0.03 | 1.82 | -3.55 - 3.62 | 0.02 | .986 |
| *DRD2 Taq1A A2/A2* ^a^ | - | - | - | - | - |
| Ethnicity: European/Caucasian | 1.04 | 0.84 | -0.61 - 2.69 | 1.24 | .216 |
| Ethnicity: Non-  European/Caucasian ^a^ | - | - | - | - | - |
| Gender: Male | 7.24 | 2.47 | 2.37 - 12.1 | 2.93 | .004 |
| Gender: Female | - | - | - | - | - |
| *DRD2 Taq1A A1/A2* * Gender ^b^ | -3.27 | 2.63 | -8.45 - 1.92 | -1.24 | .216 |
| *DRD2 Taq1A A1/A1* * Gender ^b^ | -5.11 | 2.57 | -10.17 - -0.04 | -1.98 | .048 |

Random Effects

|  | Variance | SE |
| --- | --- | --- |
| Participant (Intercept) | 11.77 | 1.73 |
| Residual | 21.51 | 1.3 |

^a^ Reference category for main effect level comparisons.

^b^ Reference categories for interactions are as indicated for main effects A2/A2)

*Supplementary Table 3. Overall model for DRD4 C521*

| Fixed Effects | Estimate | SE | 95% CI | t | p |
| --- | --- | --- | --- | --- | --- |
| Intercept | 6.05 | 0.87 | 4.33 - 7.77 | 6.92 | <.001 |
| *DRD4 C521 CC* | 0.35 | 1.03 | -1.68 - 2.38 | 0.34 | .734 |
| *DRD4 C521 CT* | 0.07 | 0.81 | -1.52 - 1.67 | 0.09 | .929 |
| *DRD4 C521 TT* ^a^ | - | - | - | - | - |
| European/Caucasian | 0.74 | 0.82 | -0.87 - 2.35 | 0.91 | .365 |
| Non-European/Caucasian ^a^ | - | - | - | - | - |
| Gender: Male | 3.83 | 0.94 | 1.99 - 5.67 | 4.09 | <.001 |
| Gender: Female ^a^ | - | - | - | - | - |
| *DRD4 C521 CC* * Gender: Male ^b^ | -1.37 | 1.49 | -4.3 - 1.57 | -0.92 | .360 |
| *DRD4 C521 CT* * Gender: Male ^b^ | -1.18 | 1.26 | -3.65 - 1.29 | -0.94 | .348 |

| Random Effects | Variance | SE |
| --- | --- | --- |
| Participant (Intercept) | 12.59 | 1.77 |
| Residual | 21.38 | 1.29 |

^a^ Reference category for main effect level comparisons.
^b^ Reference categories for interactions are as indicated for main effects.

*Supplementary Table 4. Overall model for COMT Val158*

| Fixed Effects | Estimate | SE | 95% CI | t | p |
| --- | --- | --- | --- | --- | --- |
| Intercept | 6.34 | 1.07 | 4.24 - 8.44 | 5.94 | .000 |
| *COMT Val158 GA* | -0.48 | 0.99 | -2.44 - 1.47 | -0.49 | .626 |
| *COMT Val158 AA* | 0.05 | 1.06 | -2.04 - 2.14 | 0.04 | .964 |
| *COMT Val158 GA* ^a^ | - | - | - | - | - |
| European/Caucasian | 0.77 | 0.82 | -0.84 - 2.38 | 0.94 | .348 |
| Non-European/Caucasian ^a^ | - | - | - | - | - |
| Gender: Male | 2.8 | 1.13 | 0.57 - 5.02 | 2.48 | .014 |
| Gender: Female ^a^ | - | - | - | - | - |
| *COMT Val158GA* * Gender: Male  b | 0.76 | 1.37 | -1.94 - 3.46 | 0.55 | .581 |
| *COMT Val158AA* * Gender: Male  b | -0.73 | 1.52 | -3.73 - 2.26 | -0.48 | .631 |

Random Effects

|  | Variance | SE |
| --- | --- | --- |
| Participant (Intercept) | 12.48 | 1.76 |
| Residual | 21.39 | 1.29 |

^a^ Reference category for main effect level comparisons.

^b^ Reference categories for interactions are as indicated for main effects.
